# Cancer of Pharyngoesophageal Junction: A Different Subtype From Hypopharyngeal and Cervical Esophageal Cancer?

**DOI:** 10.3389/fonc.2021.710245

**Published:** 2021-11-02

**Authors:** Xiaoyu Li, Dashan Ai, Yun Chen, Qi Liu, Jiaying Deng, Hongcheng Zhu, Ying Wang, Yue Wan, Yue Xie, Yanan Chen, Weiwei Chen, Jianhong Fan, Xiaoshen Wang, Xueguan Lu, Hongmei Ying, Xiayun He, Chaosu Hu, Kuaile Zhao

**Affiliations:** ^1^ Department of Radiation Oncology, Fudan University Shanghai Cancer Center, Shanghai, China; ^2^ Department of Radiation Oncology, Chongqing University Cancer Hospital, Chongqing, China; ^3^ Department of Oncology, Shanghai Medical College, Fudan University, Shanghai, China; ^4^ Shanghai Key Laboratory of Radiation Oncology, Shanghai, China; ^5^ Department of Oncology, The Affiliated Zhangjiagang Hospital of Soochow University, Suzhou, China; ^6^ Department of Radiation Oncology, Yancheng Third People’s Hospital, Yancheng, China; ^7^ Department of Radiation Oncology, The Affiliated Yancheng Hospital of Southeast University Medical College, Yancheng, China; ^8^ Department of Radiation Oncology, The Sixth Affiliated Hospital of Nantong University, Nantong, China; ^9^ Department of Gynaecology, Renhe Hospital, Shanghai, China

**Keywords:** esophageal neoplasms, hypopharyngeal neoplasms, lymph node metastasis, radiotherapy, survival

## Abstract

**Background:**

Squamous cell cancers in the hypopharynx (HP) and cervical esophagus (CE) are different diseases with different staging systems and treatment approaches. Pharyngoesophageal junction (PEJ) tumor involves both the hypopharynx and the cervical esophagus simultaneously, but few reports focused on PEJ tumors. This study aimed to clarify clinical characteristics and the treatment approaches of PEJ tumors.

**Patients and Methods:**

A total of 222 patients with squamous cell carcinoma in the HP, PEJ, and CE were collected between January 2008 and June 2018 in Fudan University Shanghai Cancer Center. We compared different lymph node metastatic patterns of three diseases above and the survival of different tumor locations, different lymph node metastasis, and different radiotherapy approaches.

**Results:**

For HP, PEJ, and CE cancer, the upper and middle cervical lymph node metastatic rates were 85.7%, 47.1%, and 5.8%, respectively; the lower cervical lymph node metastatic rates were 36.7%, 42.9%, and 35.0%, respectively; and the mediastinal lymph node metastatic rates were 2.0%, 72.9%, and 80.6%, respectively. The 3-year overall survival rates were 69.5% in the HP group, 52.0% in the PEJ group, and 69.6% in the CE group (*p* = 0.024). No survival differences were found between the involved-field-irradiation and elective-node-irradiation subgroups among PEJ tumors (*p* = 0.717 for OS and *p* = 0.454 for PFS, respectively).

**Conclusion:**

HP cancers had a high prevalence in all cervical lymph node metastases, while CE cancers had a lower prevalence in the cervical and mediastinal lymph node metastases. PEJ cancer had the combined metastatic patterns of both HP and CE cancers. Involved field irradiation was feasible in chemoradiotherapy for PEJ cancers.

## Introduction

As is widely recognized, squamous cell cancers in the hypopharynx (HP) and cervical esophagus (CE) are different diseases with different staging systems and treatments, although the hypopharynx and cervical esophagus are anatomically adjacent.

HP cancers are staged according to the TNM classification system of head and neck tumors, in which the head and neck lymph nodes are considered to be regional lymph nodes. Meanwhile, the TNM staging system of esophageal carcinoma is used for CE cancers and the regional lymph node area extends from the periesophageal cervical nodes in the neck to the celiac nodes (1).

Different treatment approaches should be delivered to patients with HP or CE cancers. Surgical resection is the cornerstone of the treatment of HP cancer, and partial or total laryngopharyngectomy with at least level II–IV and level VI lymph node dissection is believed to be the appropriate surgical approach. Definitive radiotherapy is used for unresectable tumors or those unfit for surgery, and primary tumor and the involved lymph nodes (local subclinical infiltration at the primary site and at the high-risk level lymph nodes included) should be given a total dose of 66–70 Gy at 2.0–2.2 Gy per fraction (2). For CE cancers, due to limited space for surgery, definitive chemoradiation is the first choice. The total dose should be 50.0–50.4 Gy at 1.8–2.0 Gy per fraction for the primary tumor, and involved regional lymph nodes with or without elective nodal regions (supraclavicular lymph nodes, etc.) and higher doses may also be appropriate (60–66 Gy) according to the NCCN guideline.

Advanced HP tumors involve the pharyngoesophageal junction in more than 30% of the cases (3), whereas 16.4%–22.5% of the CE cancers arise due to pharyngoesophageal junction (4, 5). Pharyngoesophageal junction (PEJ) tumors are defined as tumors involving simultaneously both the hypopharynx and the cervical esophagus. New problems are put forward on the tumor biology, classification systems, and treatment approaches of PEJ tumors. However, few studies on PEJ cancer were reported. Therefore, we collected the patients with squamous cell carcinoma in the HP, PEJ, and CE at the Department of Radiation Therapy of Fudan University Shanghai Center between January 2008 and June 2018, in order to clarify clinical characteristics, the treatment approaches, and the survival of PEJ tumors.

## Methods

Between January 2008 and June 2018, a total of 357 patients with squamous cell carcinoma in the HP, PEJ, and CE at the Department of Radiation Therapy of Fudan University Shanghai Cancer Center were reviewed. The study was approved by the Ethics Committee of the Fudan University Shanghai Cancer Center. All participants provided written informed consent. The inclusion criteria in this study were histologically confirmed (endoscopic biopsy or percutaneous lymph node biopsy) squamous cell carcinoma originated from HP, CE, and PEJ and treated with definitive (chemo)radiotherapy (DT ≥ 50 Gy) as initial treatment. The excluded criteria were as follows: second primary malignancy (n = 99), distant organ or lymph node metastasis at the time of diagnosis (n = 14), multiple lesions involving other esophageal segments (n = 10), unfinished RT (n = 8), and other pathological types (n = 4). A total of 222 patients with squamous cell carcinoma in the HP, PEJ, or CE matching the criteria were included in the final sample for this analysis.

### Classification and TNM Staging System

In our study, patients were divided into three groups as follows. The HP group was originated in the hypopharynx and no involvement of the esophagus, the CE group in the cervical esophagus without involvement of the hypopharynx. The PEJ group was defined as tumor involving simultaneously both the hypopharynx and the cervical esophagus.

In this study, due to lack of unified staging systems, T stages of both HP and PEJ cancers referred to the AJCC 8^th^ hypopharynx staging system (6), and CE cancer according to the AJCC 8^th^ esophagus staging system (1). As for N stages, we defined cervical and upper and middle mediastinal lymph node areas as regional lymph node areas of HP, PEJ, and CE cancer. No lymph node metastasis was defined N0 while positive lymph nodes within the regional lymph node areas was defined N+, regardless of the number and size of lymph nodes. Positive lymph nodes were defined based on CT images according to the detailed criteria below. For cervical lymph nodes, those that met at least one of the following criteria are considered positive lymph nodes: nodes with a minimal axial diameter of 11 mm or more in the subdigastric region and 10 mm or more in other lymph node-bearing regions; groups of three or more lymph nodes in a single area with a minimal axial diameter of 8 mm; lymph nodes that show irregular enhancement and that are surrounded by a rim of enhanced tumor or lymph node tissue; and lymph nodes with central necrosis or ring enhancement. For mediastinal lymph nodes, those that met at least one of the following criteria are considered positive lymph nodes: nodes with a minimal axial diameter of 10 mm or more; nodes with a minimal axial diameter of 5 mm or more in the tracheoesophageal groove area; and lymph nodes with central necrosis or ring enhancement. Any lymph node metastasis beyond the regional lymph node areas would be considered distant metastasis (M1).

**Figure 1:**
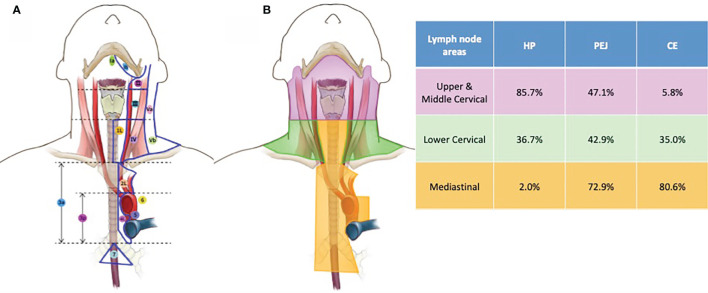
Lymph node areas **(A)** and metastatic rate **(B)** of HP, PEJ, and CE tumors. Lymph node areas, a combination of cervical and mediastinal lymph node area systems, were based on the guidelines of the Fudan University Shanghai Cancer Center. The metastatic rates of HP, PEJ, and CE tumors in the upper and middle cervical (retropharyngeal, cervical levels Ib, II, and III), lower cervical (cervical levels IV and V), and mediastinal areas (mediastinal stations 1–7). HP cancer had a high prevalence of cervical lymph node metastasis, while mediastinal lymph node metastasis was scarce. For CE cancer, lower cervical and mediastinal lymph nodes were commonly affected, with a lower prevalence of upper and middle cervical areas. Both cervical and mediastinal lymph node areas were high risk for metastasis of PEJ cancer. HP, hypopharynx; PEJ, pharyngoesophageal junction; CE, cervical esophagus.

In our study, lymph node areas were based on the guidelines of the Fudan University Shanghai Cancer Center from our previous study (7), which was a combination of traditional cervical and mediastinal lymph node area systems. Mediastinal station 1 lymph nodes were cervical paraesophageal nodes while station 2 lymph nodes were paratracheal and paraesophageal lymph nodes with paraspinal muscle as posterior border and aortic arch as inferior border. There were no differences for the borders of the rest of the lymph node areas between our guideline and the AJCC guidelines (**Figure 1**).

We also collected gross tumor volumes and planning target volumes in HP, PEJ, and CE cancers for integrated tumor staging and further analysis.

### Treatment

All patients received definitive (chemo)radiotherapy. Radiation therapy was administered with intensity-modulated radiation therapy (IMRT).

For HP cancer, gross target volume (GTV) covered primary tumor and positive lymph nodes and clinical target volume (CTV) covered both GTV and subclinical regions. In locally advanced patients, the high-risk subclinical region (CTV_high_) encompassed at least the 1-cm margin of the primary tumor and the entire subsite of the involved hypopharynx and subclinical region of lymph nodes (bilateral cervical LN levels Ib-IV as well as the lateral retropharyngeal lymph nodes for N0 patients and ipsilateral cervical LN level V for N+ patients). The low-risk subclinical region (CTV_low_) included lymph node levels II–IV and retropharyngeal LNs in the N0 neck. The planning target volumes (PTV-G, PTV-C_high_, and PTV-C_low_) were generated by adding 0.3–0.5 cm around the GTV, CTV_high_, and CTV_low_, respectively. PTV-G received a total dose of 70–70.4 Gy (5 days per week at 2.0–2.25 Gy per fraction) while PTV-C_high_ 60–63 Gy (1.75–2.0 Gy per fraction) and PTV-C_low_ 54 Gy at 1.8 Gy per fraction.

For cervical esophageal cancer, involved field irradiation (IFI) was used in 92 of 103 patients. The CTV was defined by adding 3-cm margins of the proximally and distally uninvolved esophagus without the lateral margins and lymph node regions. Elective nodal irradiation (ENI) was used in 11 patients. The CTV included not only 3-cm margins of the proximally and distally uninvolved esophagus but also supraclavicular and upper and middle mediastinal lymph node areas. The PTV of both IFI and ENI was generated by adding 1-cm margins from CTV. PTV received 50–66 Gy at 1.8–2.0 Gy per fraction.

**Figure 2:**
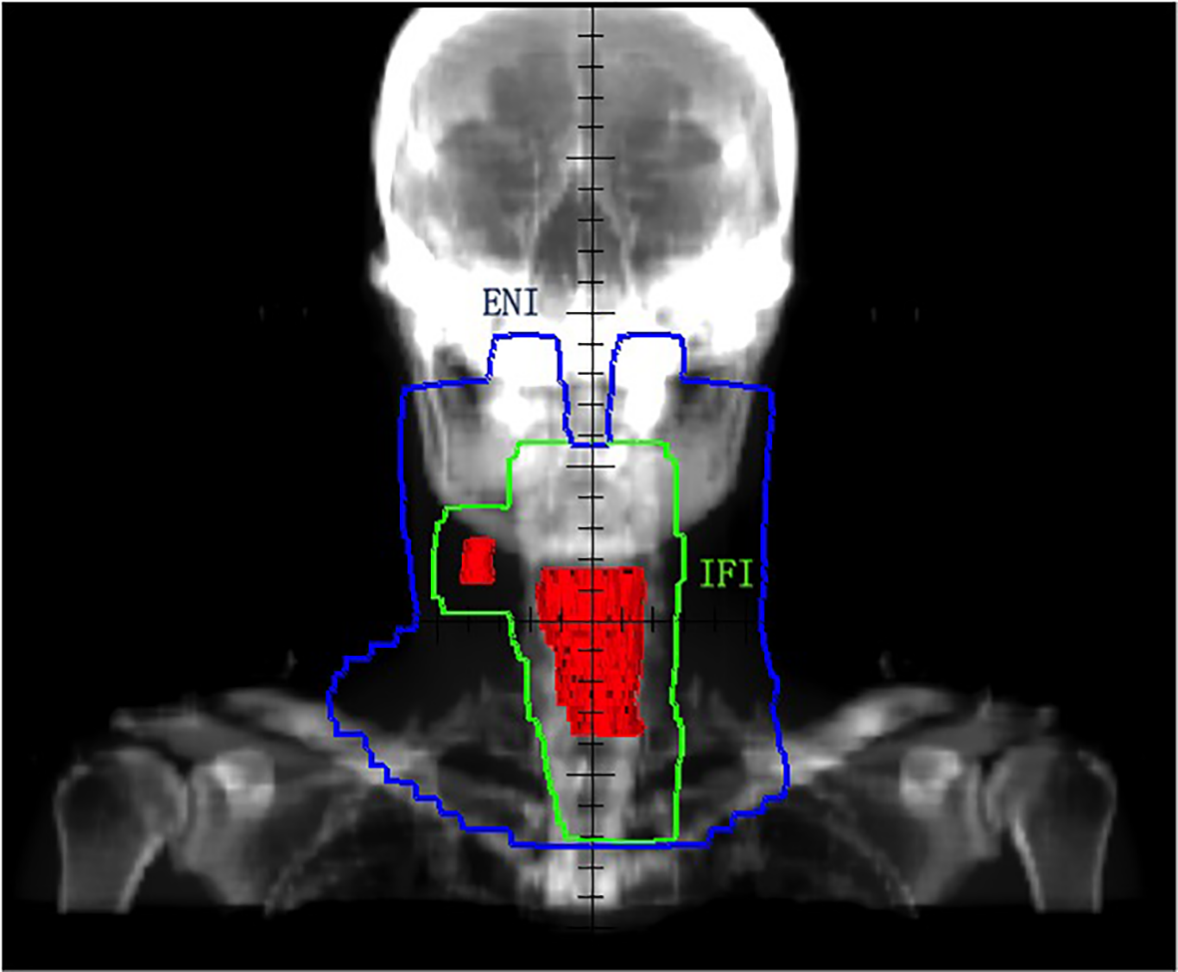
PTV of ENI and IFI for PEJ tumors. Red area for GTV (primary tumor and metastatic lymph node), green area for PTV of IFI, and blue area for PTV of ENI. GTV, gross target volume; PTV, planning target volume; ENI, elective nodal irradiation; IFI, involved field irradiation; PEJ, pharyngoesophageal junction.

For PEJ cancer, there were two different opinions on the contouring of clinical target volume (CTV) according to whether prophylactically regional nodes are areas of irradiation. One delineation adopted involved field irradiation (IFI) in 36 patients as above for CE cancer with a total dose of 61.2–66 Gy at 1.8–2.0 Gy per fraction. The other delineation underwent elective nodal irradiation (ENI) in 34 patients, which was the same with HP cancer with a total dose of 60–70 Gy at 1.8–2.25 Gy per fraction. Different delineations of PEJ cancer are shown in **Figure 2**.

For PEJ cancer, chemotherapy was performed in 62 (88.6%) patients (29 in ENI and 33 in IFI), including induction + concurrent (5 in 70, 7.1%), concurrent alone (11 in 70, 15.7%), concurrent + consolidation (36 in 70, 51.4%), and sequential chemotherapy (9 in 70, 12.9%). Chemotherapeutic regimens included paclitaxel/cisplatin (24 in 62, 38.7%), fluorouracil/cisplatin (16 in 62, 25.8%), paclitaxel/fluorouracil (18 in 62, 29.0%), and paclitaxel/cisplatin/fluorouracil (4 in 62, 6.5%).

### Endpoints and Statistical Analysis

Progression-free survival (PFS) and overall survival (OS) were defined as the time from the diagnosis to the first detection of tumor progression, and death from any cause or last follow-up, respectively. The demographic of patients and tumor characteristics were summarized with descriptive statistics. Categorical variables were analyzed by the Pearson chi-square test, and continuous variables were analyzed by the one-way ANOVA test. Survival estimation and comparison among different variables were performed using the Kaplan–Meier method. A two-sided p value of <0.05 was considered statistically significant. All statistical analyses were performed using SPSS 19.0 (SPSS, Chicago, IL, USA). Survival curves were drawn with GraphPad Prism 6.0 (GraphPad Software, San Diego, CA, USA).

## Results

### Patient and Tumor Characteristics

A total of 222 patients were enrolled, of which 49 cases were diagnosed with HP cancer, 70 cases with PEJ cancer, and 103 cases with CE cancer. All patients were predominantly male (95.9%, 91.4%, and 71.8% in the HP, PEJ, and CE groups, respectively), and patients with CE cancer were older than those with HP and PEJ cancer. Most patients were diagnosed with locally advanced disease, and nearly half or more HP and CE tumors were T3–4 stage. For PEJ tumor, 74.3% tumors were T4 stage and the rest of the tumors were T3 stage. The gross tumor volumes of PEJ cancers were much greater than those of HP or CE cancers (*p* < 0.001), indicating that PEJ cancers were more advanced than HP or CE cancers (**Table 1**).

**Table 1:** Demographic and clinical characteristics.

Features	HP N = 49	PEJ N = 70	CE N = 103	p value
Sex				<0.001
Men	47 (95.9%)	64 (91.4%)	74 (71.8%)	
Women	2 (4.1%)	6 (8.6%)	29 (28.2%)	
Age (years)				0.014
Median	59.0	58.0	61.0	
Smoking history				0.155
Never	28 (57.1%)	45 (64.3%)	51 (49.5%)	
Former/current	21 (42.9%)	25 (35.7%)	52 (50.5%)	
Drinking history				0.325
Never	21 (42.9%)	37 (52.1%)	43 (41.7%)	
Former/current	28 (57.1%)	33 (47.1%)	60 (58.3%)	
Family history of cancer				0.456
Yes	11 (22.4%)	16 (22.9%)	31 (30.1%)	
No	38 (77.6%)	54 (77.1%)	72 (69.9%)	
T stage				<0.001
T1	7 (14.3%)	NA	3 (2.9%)	
T2	20 (40.8%)	NA	18 (17.5%)	
T3	14 (28.6%)	18 (25.7%)	35 (34.0%)	
T4	8 (16.3%)	52 (74.3%)	47 (45.6%)	
Length of primary tumor (cm)				<0.001
Mean ± SD	4.99 ± 2.21	8.52 ± 2.28	5.26 ± 1.71	
N stage				0.592
N0	6 (12.2%)	8 (11.4%)	17 (16.5%)	
N+	43 (87.8%)	62 (88.6%)	86 (83.5%)	
Stage				<0.001
T1-2N0	5 (10.2%)	NA	6 (5.8%)	
T3-4N0	1 (2.0%)	8 (11.4%)	11 (10.7%)	
T1-2N+	22 (44.9%)	NA	15 (14.6%)	
T3-4N+	21 (42.9%)	62 (88.6%)	71 (68.9%)	
Gross tumor volume (mL)				<0.001
Mean ± SD	51.85 ± 31.05	68.55 ± 36.75	43.91 ± 23.71	
Differentiation grade				0.986
G1	3 (6.1%)	6 (8.6%)	8 (7.8%)	
G2	17 (34.7%)	25 (35.7%)	37 (35.9%)	
G3	21 (42.9%)	25 (35.7%)	41 (39.8%)	
Unknown	8 (16.3%)	14 (20.0%)	17 (16.5%)	
RT dose				<0.001
≥50 Gy and <66 Gy	0 (0.0%)	53 (75.7%)	99 (96.1%)	
≥66 Gy	49 (100.0%)	17 (24.3%)	4 (3.9%)	
RT strategy				<0.001
ENI	48 (98.0%)	34 (47.9%)	11 (10.7%)	
IFI	1 (2.0%)	36 (51.4%)	92 (89.3%)	
Chemotherapy				<0.001
Concurrent	30 (61.2%)	53 (75.7%)	92 (89.3%)	
Sequential	14 (28.6%)	9 (12.9%)	1 (1.0%)	
No chemotherapy	5 (10.2%)	8 (11.4%)	10 (9.7%)	

HP, hypopharynx; PEJ, pharyngoesophageal junction; CE, cervical esophagus; RT, radiation therapy; ENI, elective nodal irradiation; IFI, involved field irradiation.

In HP patients, the main invasion of hypopharyngeal substructures was the piriform sinus (85.7%), followed by posterior pharyngeal wall (65.3%) and post-cricoid region (32.7%), while that in PEJ patients was the post-cricoid region (74.3%), followed by posterior pharyngeal wall (67.1%) and piriform sinus (41.4%). Trachea (55.7%) was the most common among involved structures of PEJ tumor, followed by thyroid (17.1%) and larynx (12.9%), and for HP tumor the most common were larynx (53.1%), oropharynx (24.5%), and thyroid cartilage (14.3%) (**Table S1**).

Similar proportions of patients were diagnosed as lymph node positive with HP (87.8%), PEJ (88.6%), and CE (83.5%) tumors, but different metastatic patterns were seen in different primary tumors. A detailed positive lymph node distribution is shown in **Table 2** and **Figure 1**.

**Table 2:** Patterns of lymph node metastasis of HP, PEJ and CE cancer.

Lymph node areas	HP N = 49	PEJ N = 70	CE N = 103	p value
Cervical LN				
Retropharyngeal	11 (22.4%)	4 (5.7%)	0 (0.0%)	<0.001
Ib	1 (2.0%)	2 (2.9%)	0 (0.0%)	0.250
II	36 (73.5%)	25 (35.7%)	2 (1.9%)	<0.001
IIa	31 (63.3%)	22 (31.4%)	1 (1.0%)	<0.001
IIb	22 (44.9%)	16 (22.9%)	1 (1.0%)	<0.001
III	27 (55.1%)	19 (27.1%)	4 (3.9%)	<0.001
IV	15 (30.6%)	29 (41.4%)	36 (35.0%)	0.458
V	6 (12.2%)	2 (2.9%)	1 (1.0%)	0.004
Va	6 (12.2%)	2 (2.9%)	1 (1.0%)	0.004
Vb	2 (4.1%)	0 (0.0%)	1 (1.0%)	0.149
Mediastinal LN				
1	1 (2.0%)	47 (67.1%)	71 (68.9%)	<0.001
2	0 (0.0%)	22 (31.4%)	24 (23.3%)	<0.001
3a	0 (0.0%)	0 (0.0%)	1 (1.0%)	0.560
4	0 (0.0%)	12 (17.1%)	18 (17.5%)	0.008
5	0 (0.0%)	2 (2.9%)	4 (3.9%)	0.384
6	0 (0.0%)	0 (0.0%)	1 (1.0%)	0.560
7	0 (0.0%)	2 (2.9%)	4 (3.9%)	0.384

HP, hypopharynx; PEJ, pharyngoesophageal junction; CE, cervical esophagus; LN, lymph node areas.

For patients with HP cancer, most involved lymph nodes were located in cervical lymph node areas, especially retropharyngeal lymph nodes (22.4%) and level II (73.5%) and level III (55.1%) in the neck. Similarly, the positive rate of the upper and middle cervical lymph nodes in PEJ cancer was also very high, especially in level II (35.7%) and level III (27.1%). For patients with CE cancer, there were very few lymph node metastases in the upper and middle cervical lymph node areas (5.8% in total).

Among lower cervical lymph node regions (level IV and V in the neck), the probability of lymph node metastases of for all three cancers was similar, which was 36.7% for HP cancer, 42.9% for PEJ cancer, and 35.0% for CE cancer, respectively.

In mediastinal lymph node areas, station 1 showed the highest metastatic rate in CE and PEJ cancer (68.9% and 67.1%, respectively) followed by station 2 (23.3% for CE cancer and 31.4% for PEJ cancer, respectively) and station 4 (17.5% for CE cancer and 17.1% for PEJ cancer, respectively). Only one patient (2.0%) with HP cancer was detected mediastinal lymph node metastasis.

As for IFI or ENI subgroups among the PEJ population, there were no statistical differences between IFI and ENI subgroups of PEJ cancer in sex, age, T and N stage, primary tumor length, and gross tumor volumes, except for planning target volumes (**Table S2**).

### Survival

**Figure 3:**
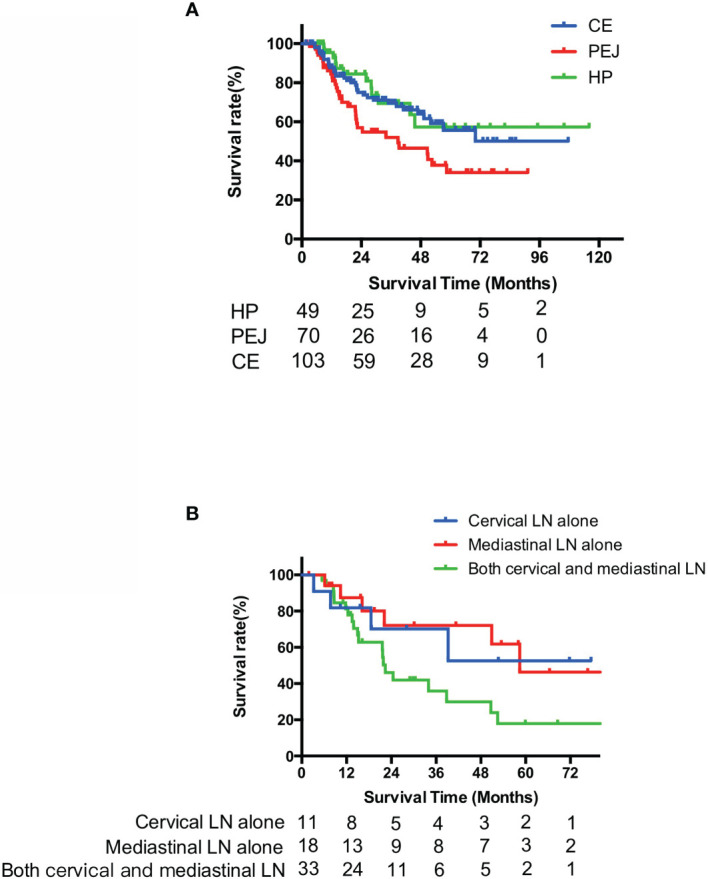
Survival curves of overall survival of HP, PEJ, and CE cancer **(A)** and PEJ cancer with different lymph node metastases **(B)**. PEJ cancer had a worse OS compared with HP cancer and CE cancer (*p* = 0.024), and in PEJ cancer, patients with both cervical and mediastinal lymph node metastases had worse OS than those with either cervical or mediastinal lymph node metastasis (*p* = 0.047). HP, hypopharynx; PEJ, pharyngoesophageal junction; CE, cervical esophagus, OS, overall survival.

There were 144 patients alive and 78 dead at the time of the last follow-up (September 30, 2019), with a median follow-up time of 30.6 months for survivors. Patients with PEJ cancer had a worse survival compared with HP cancer and CE cancer (*p* = 0.024) with a median OS of 38.8 months (95% CI, 8.7 to 68.9), while the other two groups did not reach median survival. The 1- and 3-year OS rates were 95.5% and 69.5% in the HP group, 84.4% and 52.0% in the PEJ group, and 87.8% and 69.6% in the CE group (**Figure 3A**).

For PEJ cancer, patients with both cervical and mediastinal lymph node metastasis had worse OS than those with either cervical or mediastinal lymph node metastasis (3-year OS, 70.1% for cervical only, 72.1% for mediastinal only, and 35.9% for both cervical and mediastinal, *p* = 0.047) However, no statistical difference in OS was detected between subgroups of cervical lymph node metastasis only and mediastinal lymph node metastasis only (*p* = 0.783) (**Figure 3B**).

**Figure 4:**
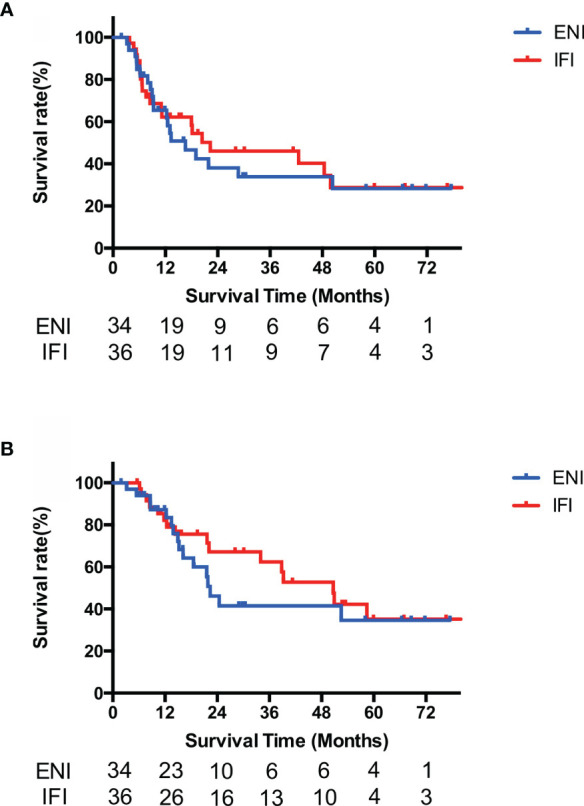
Survival curves of PFS **(A)** and OS **(B)** of PEJ cancer by different delineations. No PFS and OS differences were found between IFI and ENI subgroups among PEJ tumors (*p* = 0.717 for OS and *p* = 0.454 for PFS, respectively). PEJ, pharyngoesophageal junction; ENI, elective nodal irradiation; IFI, involved field irradiation; PFS, progression-free survival; OS, overall survival.

As for different delineation subgroups, the median PFS and OS were 16.6 months (95% CI, 9.0–24.1) and 22.4 months (95% CI, 18.2–26.5) in the ENI subgroup *versus* 22.3 months (95% CI, 0.0–47.2) and 50.6 months in the IFI subgroup (95% CI, 34.7–66.6). There were no statistical differences in OS and PFS between the two subgroups (*p* = 0.717 for OS and *p* = 0.454 for PFS, respectively) (**Figure 4**).

A total of 35 (50.0%) patients of PEJ cancer were diagnosed with recurrent, metastatic, or asynchronous second primary cancer, of which 18 cases are in the ENI group and 17 are in the IFI group. In-field recurrence accounted for more than half of failure in both groups (10 in 18 of the ENI group and 9 in 17 of the IFI group). No statistical differences were found between ENI and IFI groups in in-field recurrence rate (*p* = 0.678) and out-field metastasis rate (*p* = 0.632). Out-field regional nodal metastasis was rare, which was found in only one patient in the IFI group and none in the ENI group (**Table S3**).

## Discussion

This retrospective study is the first time to reveal the clinical characteristics, patterns of lymph node metastasis, and prognosis of different radiotherapy delineations in PEJ cancer in detail. PEJ cancer was found to be extremely aggressive, which involved not only HP and CE concurrently but also trachea, thyroid gland, and larynx at a very high incidence. In this study, we compared patterns of lymph node metastasis and survival of HP, CE, and PEJ cancers and different radiotherapy approaches for PEJ cancers.

HP cancer had a high prevalence of cervical lymph node metastasis, which are usually located in levels II, III, and IV in the neck, while mediastinal lymph node metastases were scarce. For CE cancer, lower cervical and mediastinal lymph nodes were commonly affected, with a lower prevalence of level II and level III in the neck. PEJ cancer had the combined metastatic patterns of both HP and CE cancers as a result of the involvement of HP and CE synchronously. Both cervical and superior mediastinal lymph node areas were high risk for metastasis of PEJ cancer, which was consistent with anatomic lymph node drainage patterns and previous findings in patients undergoing surgery. Martins et al. analyzed the cervical and mediastinal lymph node metastases of pharyngolaryngoesophageal tumors after neck and mediastinal node dissection and summarized other reports (8). In patients with HP cancer, the average metastasis rate was 57.6% in cervical lymph node areas and 26.1% in mediastinal lymph node areas. On the contrary, there was 25.2% on average CE cancer patients with cervical lymph node metastasis and 63.4% on average with mediastinal lymph node metastasis. PEJ cancers had both characteristics of HP and CE cancers, and the metastasis rate was 53.5% in cervical lymph node areas and 31% in mediastinal lymph node areas. The evidence based on surgical pathology was basically consistent with the trend of this study

In this study, we observed that PEJ cancer had a worse OS than HP and CE cancer. The results were in correspondence with other reports. Kim et al. demonstrated that esophageal invasion was a poor prognostic factor of 5-year disease-specific survival in patients with T4a hypopharyngeal cancer treated by surgery (9). Zhang et al. observed that hypopharyngeal extension of the CE cancer was significantly associated with 3-year PFS (*p* = 0.021) (5). Worse prognosis of PEJ cancers might be associated with more advanced diseases due to the definition of PEJ tumors and multiple lymphatic drainages of PEJ tumors.

We found that patients with synchronously positive cervical and mediastinal lymph nodes had a worse prognosis than those with either cervical or mediastinal lymph node metastasis separately in the PEJ group. Furthermore, there was no difference in prognosis between patients with PEJ cancer who had metastasis to the cervical lymph nodes alone and those with metastasis to the mediastinal lymph nodes alone. Hence, we considered that cervical and mediastinal lymph nodes were considered regional lymph nodes of PEJ cancers.

Surgery for PEJ cancers, which included PLE and reconstruction, was always accompanied by high postoperative complications and mortality rates. In a retrospective study, Wang et al. reported that postoperative mortality and morbidity rates were 9.8% and 46.3%, respectively (10). Chemoradiotherapy, as another initial treatment for PEJ cancer, had potential for larynx preservation and a lower rate of acute morbidity and mortality compared with surgery. In this study, we excluded surgical patients because different surgical methods and technical levels lead to more bias. The exclusion led to smaller differences within the study group, but at the same time more advanced patients were enrolled in the study. However, patients with PEJ cancer who underwent chemoradiotherapy had a relatively preferable survival outcome. Therefore, chemoradiotherapy could be feasible for organ preservation in patients with PEJ cancer.

Due to the low incidence of PEJ cancer, there was no consensus on the delineation of the clinical target volume. In the analysis of the two delineation modes that was performed in PEJ cancer patients, no statistical differences were found in both OS and PFS between the ENI group and IFI group, as well as the rate of in-field recurrence and out-field metastasis.

Since the radiation target volume was larger in the ENI group, patients treated with ENI showed a significantly increased risk of high-grade late toxicities than with IFI (16% *vs.* 8%, *p* = 0.047) for esophageal cancers (11). The myelosuppression occurred more severely in the ENI group than in the IFI group during concurrent chemoradiotherapy, which may result in lower completion rate in the ENI group so as to weaken the survival benefits. Moreover, patients in the IFI group might gain more opportunities of salvage radiation after regional node failure, which may lead to a better prognosis (12).

## Conclusion

PEJ cancer had the combined metastatic patterns of both HP and CE cancers as a result of the involvement of HP and CE synchronously. Both cervical and superior mediastinal lymph node areas were with high metastatic rate for PEJ cancer. Therefore, we suggest that both cervical and superior mediastinal lymph nodes should be defined as regional lymph nodes. Involved field irradiation was feasible in radical chemoradiotherapy for PEJ cancers.

## Data Availability Statement

The datasets presented in this article are not readily available because of the regulations of our center. Requests to access the datasets should be directed to KZ, kuaile_z@sina.com.

## Ethics Statement

The study was approved by the Ethics Committee of the Fudan University Shanghai Cancer Center. All participants provided written informed consent.

## Author Contributions

XYL: conceptualization, data curation, data interpretation, manuscript drafting, and manuscript editing. DA: conceptualization, formal analysis, data curation, data interpretation, manuscript drafting, and manuscript editing. YC: data curation, data interpretation, and manuscript editing. QL: data curation, data interpretation, and manuscript editing. JD: data curation, data interpretation, and manuscript editing. HZ: data curation, data interpretation, and manuscript editing. YiW: Data curation, data interpretation, and manuscript editing. YuW: data curation, data interpretation, and manuscript editing. YX: data curation, data interpretation, and manuscript editing. YNC: data curation, data interpretation, and manuscript editing. WC: data curation, data interpretation, and manuscript editing. JF: data curation, data interpretation, and manuscript editing. XW: data curation, data interpretation, and manuscript editing. XGL: data curation, data interpretation, and manuscript editing. HY: data curation, data interpretation, and manuscript editing. XH: data curation, data interpretation, and manuscript editing. CH: data curation, data interpretation, and manuscript editing. KZ: conceptualization, formal analysis, data curation, data interpretation, and manuscript editing. All authors contributed to the article and approved the submitted version.

## Funding

The research was supported by the National Key R&D Plan in China (MOST-2016YFC1303200, 2017YFC0107600), National Natural Science Foundation of China (21172043, 81872454, and 21441010), and Shanghai Anticancer Association EYAS PROJECT (SACA-CY20A04).

## Conflict of Interest

The authors declare that the research was conducted in the absence of any commercial or financial relationships that could be construed as a potential conflict of interest.

## Publisher’s Note

All claims expressed in this article are solely those of the authors and do not necessarily represent those of their affiliated organizations, or those of the publisher, the editors and the reviewers. Any product that may be evaluated in this article, or claim that may be made by its manufacturer, is not guaranteed or endorsed by the publisher.
