# Supplementary material for: Cancer of Pharyngoesophageal Junction: A Different Subtype From Hypopharyngeal and Cervical Esophageal Cancer?
Source: Front Oncol. 2021 Nov 2;11:710245. doi: 10.3389/fonc.2021.710245 (PMC8593234; doi:10.3389/fonc.2021.710245)
Supplement: Supplementary file 1 [file Table_1.docx]

| Features | HP  N=49 | PEJ  N=70 | CE  N=103 | P value |
| --- | --- | --- | --- | --- |
| Primary tumor |  |  |  |  |
| Cervical esophagus | 0  (0.0%) | 70  (100.0%) | 103  (100.0%) | <0.001 |
| Piriform sinus | 42  (85.7%) | 29  (41.4%) | 0  (0.0%) | <0.001 |
| Posterior pharyngeal wall | 32  (65.3%) | 47  (67.1%) | 0  (0.0%) | <0.001 |
| Post-cricoid region | 16  (32.7%) | 52  (74.3%) | 0  (0.0%) | <0.001 |
| Adjacent structures |  |  |  |  |
| Larynx | 26  (53.1%) | 9  (12.9%) | 0  (0.0%) | <0.001 |
| Arytenoid cartilage | 17  (34.7%) | 6  (8.6%) | 0  (0.0%) | <0.001 |
| Vocal cord | 9  (18.4%) | 5  (7.1%) | 0  (0.0%) | <0.001 |
| Ventricular band | 3  (6.1%) | 1  (1.4%) | 0  (0.0%) | 0.029 |
| Laryngeal ventricle | 0  (0.0%) | 1  (1.4%) | 0  (0.0%) | 0.336 |
| Epiglottis | 5  (10.2%) | 0  (0.0%) | 0  (0.0%) | <0.001 |
| Thyroid | 1  (2.0%) | 12  (17.1%) | 9  (8.7%) | 0.022 |
| Oropharynx | 12  (24.5%) | 4  (5.7%) | 0  (0.0%) | <0.001 |
| Trachea | 0  (0.0%) | 39  (55.7%) | 45  (43.7%) | <0.001 |
| Carotid artery | 0  (0.0%) | 0  (0.0%) | 1  (1.0%) | 0.560 |
| Thyroid cartilage | 7  (14.3%) | 2  (2.9%) | 0  (0.0%) | <0.001 |
| Cricoid cartilage | 0  (0.0%) | 2  (2.9%) | 0  (0.0%) | 0.112 |

Table S1. Adjacent structures involved by HP, PEJ and CE tumors

Abbreviations: HP, hypopharynx; PEJ, pharyngoesophageal junction; CE, cervical esophagus

|  | ENI  N=34 | IFI  N=36 | P value |
| --- | --- | --- | --- |
| Sex |  |  | 0.354 |
| Men | 30  (88.2%) | 34  (94.4%) |  |
| Women | 4  (11.8%) | 2  (5.6%) |  |
| Age (years) |  |  | 0.402 |
| Median | 59.0 | 57.0 |  |
| Length of primary tumor (cm) |  |  | 0.073 |
| Median (Range) | 9.14  (8.29-9.99) | 7.91  (7.24-8.57) |  |
| T stage |  |  | 0.075 |
| T3 | 12  (35.3%) | 6  (16.7%) |  |
| T4 | 22  (64.7%) | 30  (83.3%) |  |
| N stage |  |  | 0.932 |
| N0 | 4  (11.8%) | 4  (11.1%) |  |
| N＋ | 30  (88.2%) | 32  (88.9%) |  |

Table S2. Relations between tumor stages and different delineations for pharyngoesophageal junction cancer

Abbreviations: ENI, elective nodal irradiation; IFI, involved field irradiation

|  | ENI | IFI | P value |
| --- | --- | --- | --- |
| Total Failure | 18  (52.9%) | 17  (47.2%) | 0.632 |
| In-field Recurrence | 10  (29.4%) | 9  (25.0%) | 0.678 |
| Primary tumor | 7  (20.6%) | 8  (22.2%) | 0.868 |
| In-field lymph node | 3  (8.8%) | 1  (2.8%) | 0.276 |
| Out-field Metastasis | 6  (17.6%) | 8  (22.2%) | 0.632 |
| Out-field regional lymph node | 0  (0.0%) | 1  (2.8%) | 0.328 |
| Out-field distant lymph node | 4  (11.8%) | 3  (8.3%) | 0.632 |
| Distant organ | 3  (8.8%) | 6  (16.7%) | 0.327 |
| Second primary tumor | 3  (8.8%) | 2  (5.6%) | 0.596 |

Table S3. Failure patterns of PEJ tumor in ENI and IFI groups

Abbreviations: PEJ, pharyngoesophageal junction; ENI, elective nodal irradiation; IFI, involved field irradiation
